# Knock-Out of *DHTKD1* Alters Mitochondrial Respiration and Function, and May Represent a Novel Pathway in Cardiometabolic Disease Risk

**DOI:** 10.3389/fendo.2021.710698

**Published:** 2021-08-13

**Authors:** Chuan Wang, M. Wade Calcutt, Jane F. Ferguson

**Affiliations:** ^1^Division of Cardiovascular Medicine, Department of Medicine, Vanderbilt University Medical Center, Nashville, TN, United States; ^2^Department of Biochemistry, Mass Spectrometry Research Center, Vanderbilt University, Nashville, TN, United States

**Keywords:** mitochondrion, metabolite, alpha-aminoadipic acid, DHTKD1, respiration, Type 2 Diabetes, cardiometabolic disease

## Abstract

Cardiometabolic disease affects the majority of individuals worldwide. The metabolite α-aminoadipic acid (2-AAA) was identified as a biomarker of Type 2 Diabetes (T2D). However, the mechanisms underlying this association remain unknown. *DHTKD1*, a central gene in the 2-AAA pathway, has been linked to 2-AAA levels and metabolic phenotypes. However, relatively little is known about its function. Here we report that *DHTKD1* knock-out (KO) in HAP-1 cells leads to impaired mitochondrial structure and function. Despite impaired mitochondrial respiration and less ATP production, normal cell proliferation rate is maintained, potentially through a series of compensatory mechanisms, including increased mitochondrial content and Akt activation, p38, and ERK signaling. Common variants in *DHTKD1* associate with Type 2 Diabetes and cardiometabolic traits in large genome-wide associations studies. These findings highlight the vital role of *DHTKD1* in cellular metabolism and establish DHTKD1-mediated mitochondrial dysfunction as a potential novel pathway in cardiometabolic disease.

## Introduction

The incidence of Type 2 Diabetes (T2D) has doubled over the past twenty years and now affects over 425 million people globally (1–3), and is the 8th leading cause of death (4). T2D is associated with high mortality and incidence of comorbidities (5), including coronary artery disease, stroke, and peripheral artery disease (6). Although there is some basic understanding of disease pathophysiology, the specific causes of T2D are often unknown. Despite advances in treatment options, strategies for modulating pathogenesis before and during the onset of overt disease are still limited. Thus, a broader and more in-depth understanding of molecular pathways associated with T2D is urgently needed. In recent years, studying metabolites and their signaling pathways as disease biomarkers and mediators has become a promising avenue of discovery.

The amino acid metabolite α-aminoadipic acid (2-AAA) was identified as a novel biomarker of T2D risk in the Framingham Heart Study, where increased plasma 2-AAA in healthy individuals predicted increased future risk of T2D after 12 years of follow-up (7). 2-AAA is produced from metabolism of the essential dietary amino acid lysine (8). Lysine and 2-AAA catabolism occurs primarily in mitochondria, with 2-AAA ultimately being broken down into Acetyl Co-A before entering the TCA cycle (9). Little is known about 2-AAA function, and the mechanisms linking 2-AAA to T2D remain unknown.

The dehydrogenase E1 and transketolase domain containing 1 (*DHTKD1*) gene is a key gene in the 2-AAA catabolic pathway, encoding part of a super complex that catalyzes the conversion of 2-oxoadipate (a product of 2-AAA) to glutaryl-CoA (10, 11). In human Mendelian disorders, mutations in *DHTKD1* have been linked to 2-Aminoadipic, 2-Ketoadipic, and 2-Oxoadipic Aciduria (11, 12), as well as Charcot-Marie-Tooth Disease (13). Variation in mouse *Dhtkd1* has been found to associate with expression of the gene (eQTL) and levels of protein (pQTL) in liver, as well as with serum 2-AAA levels (14), supporting a role for DHTKD1 as a regulator of 2-AAA. Disruption of the *Dhtkd1*-2-AAA pathway in mice associates with inflammation, obesity and metabolic phenotypes (9, 15, 16). *DHTKD1* expression correlates with ATP production in mitochondria *in vitro*, and siRNA knockdown of *DHTKD1* leads to impaired mitochondrial biogenesis and increased reactive oxygen species production (17). Taken together, these data strongly suggest that *DHTKD1* plays a pivotal role in cell metabolism and may be an important regulator linking the 2-AAA pathway to metabolic disease. However, the mechanisms underlying this relationship remain unclear.

We hypothesized that *DHTKD1* plays an important role in mitochondrial energy metabolism, which may be impaired in individuals at risk of T2D. To identify mechanisms linking *DHTKD1* to metabolic dysregulation we investigated the effects of disruption of *DHTKD1* in a HAP-1 cell line. We examined the role of *DHTKD1* on mitochondrial function, and identified mechanisms linking *DHTKD1* to oxidative phosphorylation (OXPHOS) and energy metabolism.

## Material and Methods

### Cell Culture

We obtained *DHTKD1* knockout (KO) HAP1 cells and wild-type (WT) HAP1 control cells (Horizon Discovery, Dharmacon Inc). HAP1 is a human near-haploid cell line derived from the male chronic myelogenous leukemia (CML) cell line KBM-7. Cells were edited by CRISPR/Cas to have 229 bp insertion in exon 4 of *DHTKD1* (Guide RNA Sequence: TCGACAGTGAAGCGATATGG). Both WT and KO HAP1 cells were cultured in IMDM media (Gibco) with 10% fetal bovine serum and 1% penicillin/streptomycin in a humidified 5% CO_2_/95% air incubator.

### Measurement of α-Aminoadipic Acid (2-AAA)

Levels of 2-AAA in cell supernatant were quantified by liquid chromatography mass spectrometry (LCMS) at the Vanderbilt Mass Spectrometry Core. Samples were spiked with internal standard (Arginine-^15^N_4_, Sigma Aldrich), extracted with methanol, and derivatized with dansyl chloride (Sigma Aldrich) prior to analysis. The dansyl derivative of 2-AAA ([M+H]^+^ 395.1271) was measured by targeted selected ion monitoring (SIM) using a *Vanquish* ultrahigh performance liquid chromatography (UHPLC) system interfaced to a *QExactive HF* quadrupole/orbitrap mass spectrometer (Thermo Fisher Scientific). Data acquisition and quantitative spectral analysis were conducted using Thermo-Finnigan Xcaliber version 4.1 and Thermo-Finnigan LCQuan version 2.7, respectively. Calibration curves were constructed by plotting peak area ratios (2-AAA_/_Arg-^15^N_4_) against analyte concentrations for a series of 2-AAA standards. Electrospray ionization source parameters were tuned and optimized using an authentic 2-AAA reference standard (Sigma Aldrich) derivatized with dansyl chloride and desalted by solid phase extraction prior to direct liquid infusion.

### Seahorse Assay

Metabolic measurements were carried out in standard 96-well Seahorse microplates on a Seahorse XF96 analyzer, using the Mito Stress Test and Glycolysis Stress Test (Agilent). For Mito stress test, the modulators oligomycin (2uM) (TOCRIS Bioscience) as ATP synthase inhibitor, carbonyl cyanide-4 (trifluoromethoxy) phenylhydrazone (FCCP) (2uM) (Sigma-Aldrich), as mitochondrial uncoupler and a combination of rotenone (0.5uM)(Sigma-Aldrich) plus antimycin A (0.5uM) (Sigma-Aldrich) that inhibit complex I and III of the ETC were used. For the Glycolysis Stress test, the modulators glucose (10mM) (Fisher Chemical) as a source of pyruvate, oligomycin (2uM) (TOCRIS Bioscience) as ATP synthase inhibitor, and 2-deoxy-glucose (2-DG) (50mM) (Sigma), as a glycolysis inhibitor were used. The key parameters of mitochondrial function were directly measured and displayed as oxygen consumption rate (OCR). Glycolysis was measured and shown as extracellular acidification rate (ECAR). For OCR measurement, cells were incubated in unbuffered Seahorse media containing 10mM glucose, 1mM sodium pyruvate, and 2mM L-glutamine. For ECAR measurement, cells were incubated in unbuffered Seahorse media containing 2mM L-glutamine. For all experiments, 50,000 cells were seeded per well 20 hours before analysis.

### Flow Cytometry

For measurement of mitochondrial content or mitochondrial membrane potential in HAP1 cells by flow cytometry, cells were prepared and stained with MitoView Green (100nM, Biotium) or TMRE (200nM, Biotium) according to the manufacturer’s protocols.

### Mitochondrial DNA Content Analysis

The mtDNA content was analyzed by real-time PCR by absolute quantification with the following primers: mMitoF: 5’-CACCCAAGAACAGGGTTTGT-3’, mMitoR: 5’-TGGCCATGGGTATGTTGTTA -3’, mB2MF: 5’-TGCTGTCTCCATGTTTGATGTATCT -3’, and mB2MR: 5’-TCTCTGCTCCCCACCTCTAAGT -3’. Beta-2-Microglobulin (B2M) was used as an internal control. To determine mitochondrial DNA content, relative to nuclear DNA, we used the following equations (18):

(1) ΔCt=(nucDNA Ct−mtDNA Ct)

(2) $$\text{RelativemitochondrialDNAcontent}=2\times 2^{\Delta \text{CT}}$$

### Transmission Electron Microscopy (TEM)

The HAP-1 cells were washed with PBS three times and fixed in 2.5% glutaraldehyde at room temperature for 1 hour, before transfer to 4°C. TEM images were obtained using a Philips/FEI T-12 Transmission Electron Microscope at the Vanderbilt Cell Imaging Shared Resource Core according to previously described methods (19). Mitochondrial area, cell area, and cristae number were measured and quantified using ImageJ.

### Preparation of Subcellular Fractions From HAP1 Cells

Subcellular fractions were prepared as previously described, following the Basic protocol 4 (20). Briefly, to prepare the cells, WT and KO HAP1 cells were washed (60ml PBS, twice) scraped into 60-80 ml PBS and pelleted (15 min, 1000 x g, room temperature). The cell pellet was resuspended in ice-cold cell homogenization medium (150mM MgCl_2_, 10mM KCl, 10mM Tris.Cl, adjust pH to 6.7.) at a volume of medium equal to six times the volume of the pellet, and left on ice for 2 min. Cells were homogenized (Potter-Elvehjem homogenizer, five up-and-down strokes at 500 rpm) and ≥90% cell breakage confirmed by examining the homogenate under a phase-contrast microscope. Ice-cold cell homogenization medium containing 1 M sucrose (final 0.25 M) was added and mixed by gentle inversion.

To isolate the mitochondria, nuclei were first pelleted by centrifugation (5 min, 1000 x g, 4°C). The resulting supernatant was transferred to a new tube, and centrifuged (10 min, 5000 x g, 4°C) to obtain protein from the cytosol and membrane fraction. The pellet was resuspended in ∼10 ml ice-cold sucrose/Mg2+ medium using two to three gentle strokes of the pestle in a Dounce homogenizer, recentrifuged (10 min, 5000 x g, 4°C), and resuspended in 2 to 3 ml ice-cold mitochondrial suspension medium I.

### Western Blot

DHTDK1 antibody (Proteintech, 1:1000), β-Actin antibody (Proteintech, 1:5000), Total OXPHOS antibody (Abcam, 1:1000, this OXPHOS antibody contains five mouse mAbs, one each against Complex I NDUFB8, Complex II SDHB, Complex III UQCRC2, Complex IV MTCO1, and Complex V ATP5A), COX IV antibody (Cell Signaling, 1:1000), anti-phospho AKT (Cell Signaling, Ser473, 1:1000), anti-phospho AKT (Cell Signaling, Thr 308,1:1000), anti-AKT (Cell Signaling, 1:1000), anti-phospho ERK1/2 (Cell Signaling, 1:2000), anti-ERK1/2 (Cell Signaling, 1:1000), anti-phospho p38 (Cell Signaling, 1:2000), anti-p38 (Cell Signaling, 1:1000), anti-phospho JNK (Cell Signaling, 1:1000) and anti-JNK (Cell Signaling, 1:1000) were diluted in 5% milk, Tris-buffered saline TBS, and 0.1% Tween 20. Secondary antibodies (Sigma, Anti-Rabbit IgG, A3687; Sigma, Anti-Mouse IgG, A3562) were then used at 1:20000 dilution. Total protein was quantified prior to loading, and equal amounts of protein (30µg) loaded per lane. Bands were imaged by fluorescence, and relative protein abundance estimated using ImageJ.

### Cell Proliferation Rate Measurement

Both HAP1 WT and KO cells were seeded at a density of 10^4/well in 6-well plates. Cells were detached by trypsin EDTA (ATCC) and the cell density in each well was counted separately over the following 5 days.

### MTT Assay

Cell proliferation and viability was confirmed by the MTT (3-(4,5-dimethylthiazol-2-yl)-2,5-diphenyl tetrazolium bromide) assay (Sigma-Aldrich). Both HAP1 WT and KO cells were seeded at a density of 5000 cells/well in 96-well plates. Cell viability was assessed after the cells were incubated for 24h, 48h, 72h, and 96h.

### Human Genetic Analysis

We queried single nucleotide polymorphisms (SNPs) mapping to the *DHTKD1* gene (coding region ±50kb) for their association with cardiometabolic traits using genome-wide association study (GWAS) summary statistics data available through the Common Metabolic Diseases Knowledge Portal (https://hugeamp.org/). Phenotypes included Type 2 Diabetes (N=1,183,150), random blood glucose (N=468,542), hemoglobin A1C (HbA1C; N=277,643) and Coronary Artery Disease (N=1,479,550).

### Statistical Analysis

Statistical analysis was completed in the GraphPad Prism 9.0 package using unpaired t-tests, assuming equal variances. A threshold of P < 0.05 was considered statistically significant.

## Results

### Absence of DHTKD1 Decreases Mitochondrial Respiration in HAP-1 Cells

**Figure 1:**
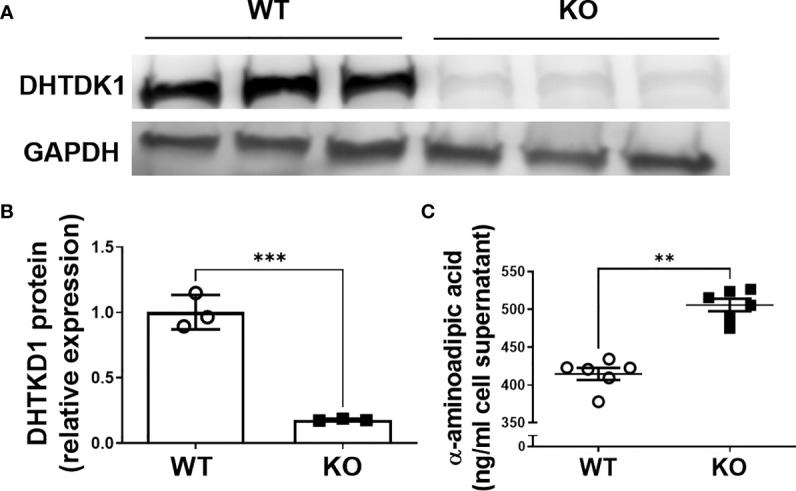
Verification of DHTKD1 KO in HAP-1 cells. **(A)**. Total protein was isolated and the expression of DHTKD1 and GAPDH was detected by Western blotting, n=3 (Triplicates for HAP-1 WT and KO respectively). Consistent with expectation, there was almost no expression of DHTKD1 protein in KO cells. **(B)** Quantification of Western blotting data in panel **(A)** normalized to GAPDH. ***P < 0.001 Data are presented as the mean ± standard error of the mean. **(C)** Levels of 2-AAA were measured in the supernatant from equal numbers of WT and KO cells (cells were seeded at the same density and supernatant collected after overnight incubation). There was significantly more (P=0.002) 2-AAA secretion from KO cells, consistent with a reduction in DHTKD1-mediated catabolism of 2-AAA. **P < 0.01.

**Figure 2:**
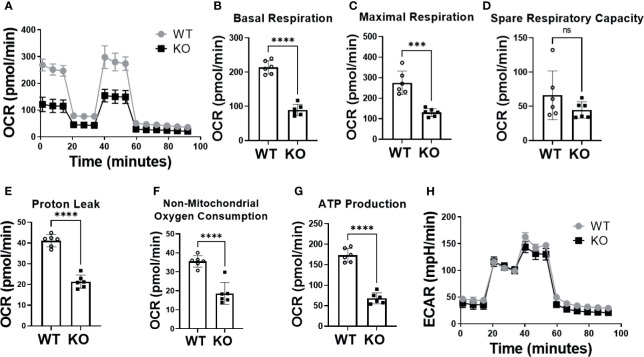
Absence of DHTKD1 decreased mitochondrial respiration. **(A)** Overall oxygen consumption rate (OCR) was significantly lower in KO cells compared to WT (P<0.05) including differences in **(B)** Basal Respiration (P<0.0001); **(C)** Maximal Respiration (P=0.0002); **(D)** Spare Respiratory Capacity (P=0.1912); **(E)** Proton Leak (P<0.0001); **(F)** Non-mitochondrial Oxygen Consumption (P<0.0001) and **(G)** ATP Production (P<0.0001). **(H)** There were no differences in glycolysis, measured by the extracellular acidification rate (ECAR). Differences assessed by T test ***P < 0.001, ****P < 0.0001. Each assay (n=6 for technical replicates) was repeated for 3 times, and OCR and ECAR values showed in each panel were averaged. ns, Not Statistically Significant.

To verify successful knockdown of DHTKD1, we measured DHTKD1 protein levels in WT and KO cells by Western blot and confirmed a significant reduction in DHTKD1 protein in the KO cells (**Figures 1A, B**). We observed no obvious differences in overall cell morphology between WT and KO cells. There was a significant increase (P=0.002) in 2-AAA in the media of KO cells compared with WT (**Figure 1C**), consistent with absence of DHTKD1 activity to catabolize 2-AAA. To investigate the effects of *DHTKD1* disruption on cell respiration, we performed seahorse assay on WT and KO cells. The KO cells had significantly lower OCR when compared to WT cells (**Figure 2A**). Further, basal respiration, maximal respiration, proton leak, non-mitochondrial oxygen consumption and ATP production were all significantly decreased in KO cells (**Figures 2B–G**; all P<0.05 but panel D). There were no differences in extracellular acidification rate (ECAR) between WT and KO cells (**Figure 2H**), suggesting no differences in glycolysis.

### DHTKD1 KO Decreases Electron Transport Chain Related Proteins Both in Mitochondria and Cytosol

**Figure 3:**
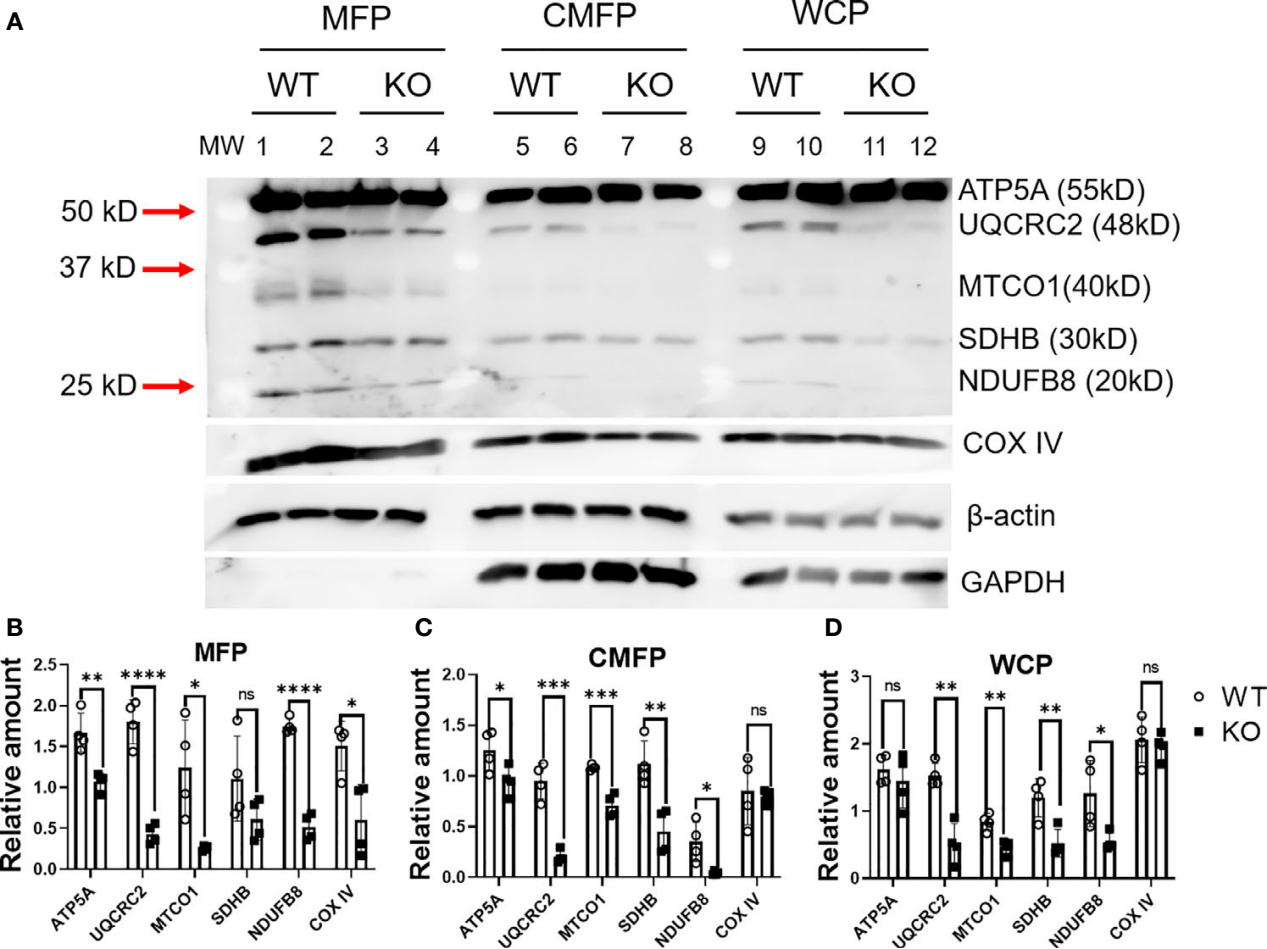
Absence of DHTKD1 leads to altered protein expression in mitochondria and cytosol. **(A)** Expression of key proteins were altered in DKTHD1 KO cells. Lane 1-4, protein from mitochondrial fraction; Lane 5-8, protein from cytosol and membrane fraction, Lane 9-12, protein from whole cell lysate. **(B–D)** Western blot quantification of proteins from MFP, CMFP, and WCP, normalized to β-actin, n=4 represents loading of four independent cell lysates. Differences assessed by T test *P < 0.05, **P < 0.01, ***P < 0.001, ****P < 0.0001. Data are presented as the mean ± standard error of the mean. ns, Not Statistically Significant.

To gain insight into the mechanisms that could explain differences in mitochondrial respiration between KO and WT cells, we assessed effects on oxidative phosphorylation. Levels of OXPHOS proteins (total OXPHOS antibody: NDUF88, SDHB, MTCO1, UQCRC2, ATP5A), and COX IV were determined by Western blot in WT and KO cells. Proteins were quantified using ImageJ and normalized to β-actin levels before analysis. We found that multiple proteins from complex I to complex V were significantly decreased in KO cells (UQCRC2, MTC01, SDHB, NDUFB8, P<0.05) with a trend towards reduction in all proteins measured (**Figure 3A**, lane 9-12; **Figure 3D**). Next, we prepared subcellular fractions of mitochondrial and cytosolic proteins from WT and KO cells and determined the related expression level of proteins in the electron transport chain. We found that ATP5A, UQCRC2, MTCO1, NDUFB8, and COX IV were significantly (P<0.05) decreased in the mitochondrial fraction (MFP), with a trend towards a decrease in SDHB (P ≤ 0.15) (**Figure 3A**, lane 1-4; **Figure 3B**). Moreover, ATP5A, UQCRC2, MTC01, SDHB, and NDUFB8 were significantly decreased in the cytosolic and membrane fraction (CMFP), while COX IV did not change (P=0.7556) (**Figure 3A**, lane 5-8; **Figure 3C**). GAPDH, a housekeeping gene that normally expresses in cytosol, was not detected in MFP, indicating the success of separating subcellular fractions of mitochondrial and cytosolic proteins (**Figure 3A** lane 1-12).

### DHTKD1 KO Alters Mitochondrial Morphology

**Figure 4:**
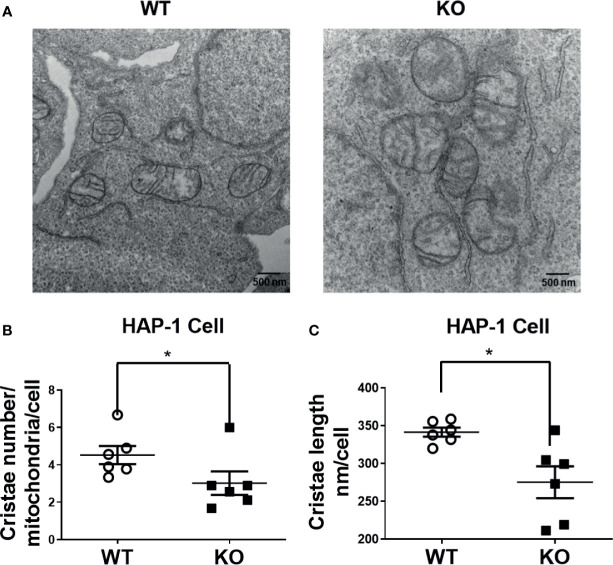
Altered cristae number and length in HAP-1 KO cells. **(A)** Representative images of mitochondria from HAP1 cells were captured by transmission electron microscope. **(B)** Cristae number were counted in 9 mitochondria and averaged in 6 representative cells each for WT and KO. **(C)** 15 mitochondrial cristae length were measured and averaged in 6 representative cells each for WT and KO by ImageJ. *P < 0.05

To examine whether DHTKD1 influences mitochondrial morphology, we captured high magnification images of mitochondria in WT and KO cells by transmission electron microscopy (**Figure 4A**). Both the number and length of cristae per cell were significantly decreased in KO cells (**Figures 4B, C**). Taken together, these data suggest that lower mitochondrial respiration in *DHTKD1* KO cells may be attributable to reduced expression of electron transport chain proteins in mitochondria and impaired cristae formation.

### DHTKD1 KO Cells Have Increased Mitochondrial Content and No Impairment in Cell Proliferation

**Figure 5:**
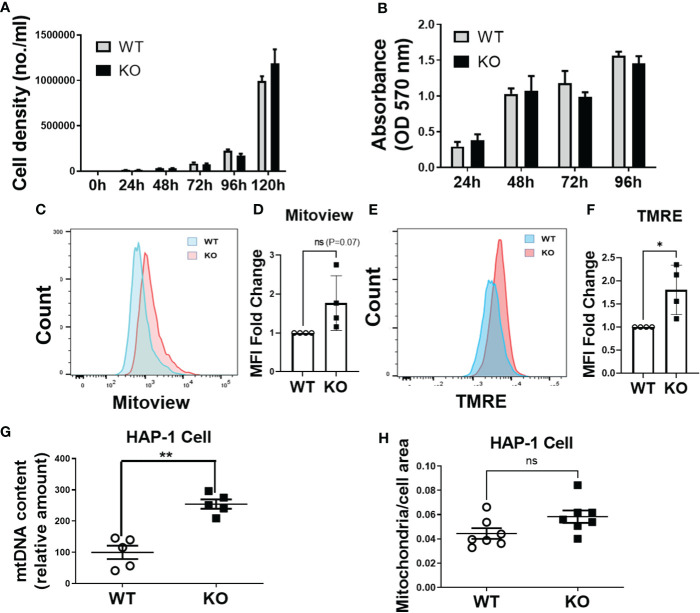
Mitochondrial content is increased in DHTKD1 KO cells, and may partially compensate for defects in mitochondrial structure. **(A)** Equal numbers of WT and KO cells were plated, and counted over 5 days. There was no apparent difference in cell proliferation, n=6. **(B)** Cell viability was determined by MTT, n=6 to confirm there were no differences in cell proliferation. **(C, E)** DHTKD1 KO cells have higher mitochondrial content and higher membrane potential than WT cells. **(D, F)** Data from representative flow cytometry were quantified by mean fluorescence intensity (MFI). **(G)** qPCR of mitochondrial DNA from both WT and KO HAP-1 cells showed higher expression in KO, (n=5, represents five independent DNA isolation). **(H)** Mitochondrial area and the whole cell area were measured in ImageJ, with slightly higher ratio of mitochondria to cell area in KO cells (P=0.05). *P < 0.05, **P < 0.01, data are presented as the mean ± standard error of the mean. ns, Not Statistically Significant.

Given our observations that *DHTKD1* KO caused a significant reduction in mitochondrial respiration, we were interested in potential effects on cell proliferation. However, we found no differences in the cellular proliferation rate between WT and KO cells when examining cell counts (**Figure 5A**) or by MTT assay (**Figure 5B**). Based on this observation, we hypothesized that there must exist mechanisms that provide compensatory responses to a reduction in mitochondrial function in KO cells. We performed flow cytometry analysis of WT and KO cells to characterize mitochondrial content. The intensity of mitoview in KO cells showed higher trend than that in WT cells, suggesting increased mitochondrial content (**Figures 5C, D**). Similarly, the intensity of TMRE was also higher in KO cells, indicating that KO cells have higher cell membrane potential (**Figures 5E, F**). To cross verify our results, we isolated mitochondrial DNA (mtDNA) from the cells. Consistent with the flow cytometry results, KO cells contained significantly more mtDNA compared to WT cells (**Figure 5G**). High magnification images of mitochondria captured by transmission electron microscopy (**Figure 4A**), showed that the ratio of mitochondrial area to whole-cell area was higher in KO cells compared to WT (**Figure 5H**), confirming increased mitochondrial content in *DHTKD1* KO cells. Taken together, these data suggest that *DHTKD1* KO cells may partially compensate for reduced mitochondrial function by increasing their mitochondrial content.

### Phosphorylation of Mitogen-Activated Protein Kinase (MAPK) and Phosphoinositide 3-Kinases (PI3Ks) Pathway Proteins Is Increased in DHTKD1 KO Cells

**Figure 6:**
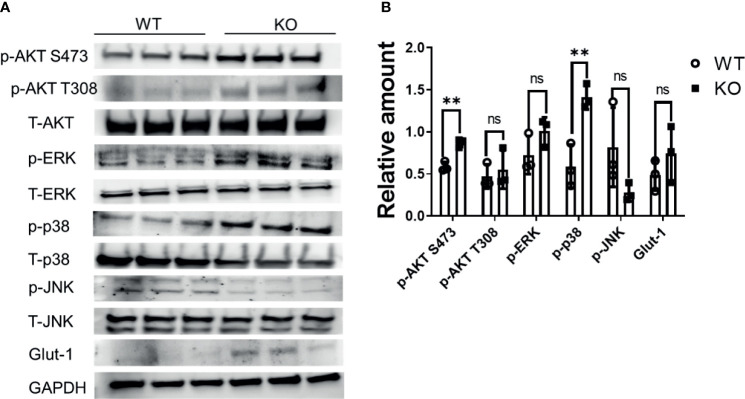
MAPK signaling is increased in DHTKD1 KO cells. **(A)** Total protein was isolated from WT and KO cells (n=3, represents three independent cell lysates), and detected by Western blotting. KO cells had higher phosphorylation of AKT, ERK, and p38, and higher expression of Glut1. **(B)** Western blot quantification of AKT, ERK, p38, JNK, and Glut-1 (p-AKT, p-p38, p-ERK, and p-JNK were normalized to T-AKT, T-p38, T-ERK, and T-JNK respectively; Glut1 was normalized to GAPDH). **P < 0.01, data are presented as the mean ± standard error of the mean. ns, Not Statistically Significant.

To further assess potential compensatory mechanisms relating to cell proliferation, we determined the basal phosphorylation level of kinases in the MAPK (p38, ERK, JNK) and PI3K (AKT) pathway. The basal phosphorylation level of p38, and AKT S473, but not JNK kinase, were significantly increased in KO cells (P<0.002) (**Figures 6A, B**). Activation of p38, and AKT signaling may compensate for impaired mitochondrial function in *DHTKD1* KO cells.

### Common Variants in DHTKD1 Associate With Type 2 Diabetes

To assess the potential impact of variants in *DHTKD1* in human disease, we queried publicly available genome-wide association data from up to 1.4 million individuals through the Common Metabolic Diseases Knowledge Portal. We identified several SNPs in *DHTKD1* that were highly significantly associated with cardiometabolic traits, including Type 2 Diabetes (top SNP rs11257655, p=2.4x10^-61^), glucose (top SNP rs11257655, p=5.x10^-18^), HbA1C (top SNP rs12221133, p=3.0x10^-12^) and Coronary Artery Disease (top SNP rs7068966, p=1.9x10^-11^).

## Discussion

**Figure 7:**
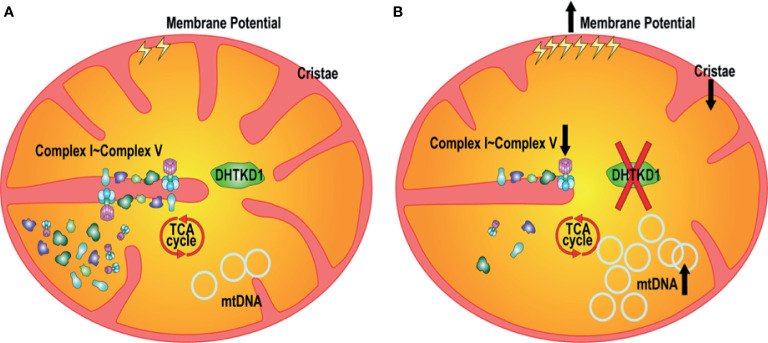
Proposed mechanisms linking disruption of DHTKD1 to altered mitochondrial function. **(A)** DHTKD1 is an important protein in mitochondrial metabolism. **(B)** DHTKD1 KO causes impairments in mitochondrial cristae structure, leading to impaired mitochondrial respiration. Absence of DHTKD1 also increases cell mitochondrial membrane potential, mitochondrial content, mtDNA content, and the ratio of mitochondrial area to the total cell area, which may compensate for the impaired function and permit maintenance of normal cell proliferation.

Understanding the mechanisms underlying novel disease associations may allow for increased understanding of disease pathophysiology. Previous work identified 2-AAA as a metabolite of interest for type 2 diabetes (7) and atherosclerosis (21), however the molecular mechanisms are unknown. It has been found that at the cellular level, diabetes is closely related with the structure and function of mitochondria (22, 23). Here we identified *DHTKD1* as a key protein of interest, due to its involvement in the 2-AAA pathway and examined the relationship between *DHTKD1* and mitochondrial function. We found that knock out of *DHTKD1* resulted in decreased mitochondrial respiration with no changes in glycolytic function. Further, we discovered that knock out of *DHTKD1* also led to reduced expression of essential proteins of the electron transport chain (NDUFB8, MTCO1, UQCRC2, COX IV, ATP5A), particularly in mitochondria. Moreover, the cristae number and the average length of cristae per cell were reduced in KO cells. There were no differences in cell proliferation rates between WT and KO cells. *DHTKD1* knock out led to increased phosphorylated AKT S473 and p38. Further, *DHTKD1* knock out increased cell mitochondrial membrane potential, mitochondrial content, mtDNA expression, and mitochondrial area. These findings demonstrate that DHTKD1 regulates morphology and function of mitochondria, and that disruption of DHTKD1 leads to impairments in mitochondrial energy metabolism, as summarized in **Figure 7**.

Here we focused on *DHTKD1* as a primary regulator of the 2-AAA pathway, because of abundant data from human, cell, and animal models implicating this gene in 2-AAA-related dysfunction (11, 14). Given the known role of DHTKD1 in mitochondria (17, 24), we hypothesized that disruption of *DHTKD1* would affect mitochondrial metabolism. Consistent with our hypothesis, we found that mitochondrial respiration was significantly impaired in cells lacking *DHTKD1*, with a reduction in both basal and maximal respiration, and significantly lower production of ATP. There was no effect on glycolysis, confirming that *DHTKD1* is not a regulator of glucose metabolism *per se*. These data suggest that impairments in DHTKD1 function cause a reduction in metabolic rate. Impaired mitochondrial energy metabolism is linked to development of diabetes and obesity (22, 23, 25), however whether impairments in DHTKD1 function predispose to the development of diabetes, obesity and cardiometabolic disease through modulation of mitochondrial energy metabolism remains to be determined.

Having observed that DHTKD1 KO led to reduced mitochondrial energy metabolism by seahorse assay, we were interested in understanding the mechanisms underlying this observation. We examined the structure of the mitochondria, and observed significant impairments in the inner mitochondrial membrane, with both the average length and the average number of cristae per mitochondria significantly decreased in *DHTKD1* KO cells. DHTKD1 is a close protein homolog of oxoglutarate dehydrogenase (OGDH), and the proteins are thought to interact directly to form a super complex, potentially also with dihydrolipoyl dehydrogenase (DLD) and dihydrolipoyl succinyltransferase (DLST) (10, 26). Disruption of DHTKD1 may thus affect one or more protein complexes in the pathway, with potential functions beyond catabolism of 2-AAA.

Having observed reduced mitochondrial respiration in *DHTKD1* KO cells, we hypothesized that this could be related to impairments in oxidative phosphorylation (27). We found that levels of OXPHOS proteins were significantly reduced in KO cells. We separated total cellular proteins into cytosol/membrane fraction and mitochondrial fraction and found that OXPHOS proteins were dramatically decreased in the mitochondrial fraction, as well as in the cytosol/membrane fraction, with significant reduction in COXIV in mitochondria. These data suggest that impaired mitochondrial respiration in *DHTKD1* KO cells could be attributable to reduced expression of electron transport chain proteins, or impaired ability to transport key proteins into mitochondria.

Absence of *DHTKD1* impaired mitochondrial respiration. Since mitochondrial respiration can be rate limiting in cell proliferation, we hypothesized KO of *DHTKD1* might lead to decreased cell proliferation rate. Silencing of *DHTKD1* has previously been reported to lead to decreased proliferation in hepatic cells (17). However, to our surprise, we observed no differences in the cell proliferation rate between WT and KO cells, suggesting the existence of compensatory mechanisms. To gain insight into these potential mechanisms, we examined mitochondrial content in WT and KO cells. We observed higher mitochondrial content and evidence for increased mitochondrial activation in the KO cells based on flow cytometry measurements. We confirmed this observation through measurement of mtDNA content, where KO cells had a higher amount of mitochondrial DNA, suggestive of a greater number of mitochondria. Because mitochondria in cells are continuously remodeled by fusion and fission of the organelles (28, 29), it is difficult to count the mitochondrial number in cells precisely. Thus, we measured the ratio of mitochondrial area to the whole cell area and found that this was greater in the KO cells. These data are consistent with *DHTKD1* KO leading to upregulation of the production of mitochondria, and higher mitochondrial activity, likely as an attempt to compensate for impaired mitochondrial function. Since both MAPK and PI3K pathways are involved in cell division and proliferation, we examined basal phosphorylation level of proteins in the MAPK pathway (p38, ERK,JNK) and in PI3K pathway (AKT). We found that p38 and AKT were more phosphorylated in KO cells. These data may point towards potential mechanisms that compensate for the absence of DHTKD1 and suggest that absence of DHTKD1 does not always lead to decreased proliferation in all cells.

Given the importance of *DHTKD1* in mitochondrial function, we hypothesized that common variants in *DHTKD1* might associate with cardiometabolic disease. Genome-wide association data confirmed a strong association between SNPs in the *DHTKD1* region and Type 2 Diabetes, in addition to glucose and HbA1c. We also observed an association with Coronary Artery Disease, suggesting that the effects of disruption in *DHTKD1* function may affect overall risk of cardiometabolic disease.

Human studies have clearly linked elevated 2-AAA to worse outcomes, including increased diabetes incidence (7), and higher degree of coronary artery calcification (21). However, data in mice are conflicting, where 2-AAA has been suggested to be protective against diet-induced obesity (16). We consider two possibilities for these discrepancies. First, it is possible that 2-AAA has differing effects in mice and humans, and that using a mouse model to study the relevance of 2-AAA to human disease is inappropriate. Our data suggest that absence of *DHTKD1* in human cells leads to impaired mitochondrial respiration. Disruption of *Dhtkd1* in mice has been reported to lead to lower serum insulin in *Dhtkd1* -/- mice compared with WT on re-feeding (16). However, this same mouse model has been used as a model of Charcot Marie Tooth disease, where higher serum insulin was reported in *Dhtkd1* -/- mice compared with WT in both the fasted and re-fed state (15). Further, 2-AAA treatment was reported to either increase (15) or decrease (16) insulin in the same mouse model. Thus, data are conflicting, even considering data from the same mouse model and research group. The severe effects of *Dhtkd1* knockout on motor and neurological function in mice (15) may make this an inappropriate model for the study of metabolic disease. Second, we consider there may be differences in the effects of 2-AAA depending on disease state. We hypothesize that 2-AAA may be up-regulated in response to metabolic stress, such as may occur during onset of disease. Elevation of 2-AAA in a pre-disease state may temporarily improve metabolic function, potentially through up-regulation of insulin secretion in addition to effects on respiration, but sustained elevation of 2-AAA may not fully compensate and may itself be deleterious. This hypothesis remains to be tested in future human-centric studies.

Our study has considerable strengths, as well as some limitations. We used a CRISPR/Cas-edited HAP1 human cell line to study *DHTKD1* knock-out. HAP1 cells have considerable utility for genetic research, and have been successfully used for examination of mitochondrial phenotypes (30, 31), as well as cardiometabolic traits (32, 33), making them an informative model for understanding the general role of *DHTKD1* in mitochondrial function. However, this chronic myeloid leukemia-derived cell line may not recapitulate all phenotypes of direct relevance to the pathogenesis of diabetes or cardiometabolic disease. Future studies are required to determine whether disruption of *DHTKD1* causes similar phenotypes across multiple cell types, particularly those of high relevance to cardiometabolic disease.

In conclusion, we examined the role of *DHTKD1* in mitochondrial metabolism, and identified a novel role for *DHTKD1* as a regulator of mitochondrial respiration, through effects on mitochondrial structure and function. Human genetic data further highlight the relevance of DHTKD1 in diabetes and cardiometabolic disease. Taken together, these data may support a role for altered mitochondrial energy metabolism and dysregulation of the 2-AAA-DHTKD1 pathway as an early driver of diabetes and cardiometabolic disease.

## Data Availability Statement

The original contributions presented in the study are included in the article/supplementary material. Further inquiries can be directed to the corresponding author.

## Author Contributions

JF and CW contributed to conception and design of the study. CW and MC performed experiments. CW wrote the first draft of the manuscript. All authors contributed to the article and approved the submitted version.

## Funding

The project was supported by a grant from the NIH (R01DK117144, PI: JF).

## Conflict of Interest

The authors declare that the research was conducted in the absence of any commercial or financial relationships that could be construed as a potential conflict of interest.

## Publisher’s Note

All claims expressed in this article are solely those of the authors and do not necessarily represent those of their affiliated organizations, or those of the publisher, the editors and the reviewers. Any product that may be evaluated in this article, or claim that may be made by its manufacturer, is not guaranteed or endorsed by the publisher.
